# Development of a national outpatient parenteral antimicrobial therapy (OPAT) pathway for skin and soft tissue infections in Scotland

**DOI:** 10.1093/jacamr/dlag227

**Published:** 2026-11-05

**Authors:** R A Seaton, C Vallance, B White, R Esiovwa, F Robb, G Mooney, D Rigby, C L Mackintosh, A MacDonald, F Kerr

**Affiliations:** Infection Unit and OPAT Service, Queen Elizabeth University Hospital, 1345 Govan Road, Glasgow G51 4TF, UK; Scottish Antimicrobial Prescribing Group, Healthcare Improvement Scotland, Delta House, 50 West Nile street, Glasgow G12 NP6, UK; Infection Unit and OPAT Service, Queen Elizabeth University Hospital, 1345 Govan Road, Glasgow G51 4TF, UK; Infection Unit and OPAT Service, Queen Elizabeth University Hospital, 1345 Govan Road, Glasgow G51 4TF, UK; Scottish Antimicrobial Prescribing Group, Healthcare Improvement Scotland, Delta House, 50 West Nile street, Glasgow G12 NP6, UK; Infection Unit and OPAT Service, Queen Elizabeth University Hospital, 1345 Govan Road, Glasgow G51 4TF, UK; Scottish Antimicrobial Prescribing Group, Healthcare Improvement Scotland, Delta House, 50 West Nile street, Glasgow G12 NP6, UK; National Improvement Advisor Unscheduled Care, National Centre for Sustainable Delivery, NHS Golden Jubilee National Hospital, Glasgow, UK; General Practitioner, Langabhat Medical Practice, Leurbost, Isle of Lewis HS2 9JP, UK; Regional Infectious Disease Unit, NHS Lothian Western General Hospital, Crewe Road South, Edinburgh EH4 2XU, UK; Pharmacy Department, Raigmore Hospital, Inverness IV2 3UJ, UK; Scottish Antimicrobial Prescribing Group, Healthcare Improvement Scotland, Delta House, 50 West Nile street, Glasgow G12 NP6, UK

## Abstract

**Background and objectives:**

The Scottish Antimicrobial Prescribing Group (SAPG) formed a multidisciplinary outpatient parenteral antimicrobial therapy (OPAT) working group in September 2020 to develop a national consensus on OPAT treatments and to support service development.

**Methods:**

The development of a national OPAT skin and soft tissue (SSTI) clinical pathway for adults was identified as a key initiative to support admission avoidance. The pathway was modelled on an established pathway in one health board and agreed by consensus with representation from 12 of the 14 territorial Scottish health boards. A survey to assess pathway uptake was conducted in February 2025, and a follow-up survey of boards that had not adopted the pathway was conducted in April 2026.

**Results:**

IV ceftriaxone was agreed by the SAPG as initial OPAT SSTI therapy, with daptomycin or teicoplanin as alternatives, and with dalbavancin to support care of vulnerable adults or if daily IV therapy was not possible. The pathway was circulated to all health boards in June 2022. By April 2026 the pathway had been adopted by 11 (2 island and 9 mainland) health boards across 28 separate acute services including OPAT, hospital at home, emergency departments and ambulatory care units. The remaining three health boards reported OPAT activity but without formal adoption of the pathway.

**Conclusions:**

A national OPAT SSTI pathway has been successfully developed and rolled out across approximately 91% of the adult population in Scotland, including remote and rural areas. Further work to evaluate use and wider rollout is ongoing.

## Introduction

The Scottish Antimicrobial Prescribing Group (SAPG) oversees antimicrobial stewardship (AMS) activities across Scotland,^1^ where outpatient parenteral antimicrobial therapy (OPAT) has been a key infection specialist-led AMS service supporting early hospital discharge and providing a cost-effective alternative to hospital admission for patients with a range of infections.^2–6^ Skin and soft tissue infections (SSTIs) are the most frequently managed OPAT conditions in the UK.^7^ Nurse-led patient group directions (PGDs) have enhanced AMS by demonstrating shorter durations of therapy and earlier IV-to-oral switch (IVOS) in some services.^8,9^ Variation in the delivery of infection admission avoidance strategies by health boards was observed during COVID-19. In response SAPG convened a multidisciplinary group (SAPG OPAT) to develop a national consensus on OPAT treatment pathways. The development of a national OPAT SSTI clinical pathway is described.

## Materials and methods

The SAPG OPAT group was formed in September 2020 and comprised multidisciplinary (medical infection specialists, general practice, OPAT nursing and antimicrobial pharmacy) representation from across 12 of 14 health boards. Meetings were virtual, occurring 2- to 3-monthly with reporting to SAPG. It was agreed to prioritize pathway development for OPAT SSTI management with a focus on remote and rural populations. The pathway was developed through a process of discussion and agreement using an already well-established clinical pathway in one health board.^8,9^ Key AMS considerations including selection of IV agents and timing of IVOS were incorporated. Challenges of equity with respect to harder-to-reach adults, including those in remote and rural settings and people who inject drugs (PWIDs), were considered.^3,5,10^

An electronic (Microsoft forms) non-anonymized OPAT survey of service staffing, facilities and challenges, and including availability and use of an SSTI pathway, OPAT provision for PWIDs and availability of OPAT independent (non-medical) prescribers, was designed. It was circulated in February 2025 to all 14 territorial health boards (11 mainland and 3 island).

A follow-up survey was sent to contacts who had not implemented the pathway or who did not have an OPAT service in place in April 2026. OPAT leads were contacted to specify which clinical services were utilizing the pathway.

## Results

SAPG OPAT included representation from 12 health boards (10 of the 11 mainland boards and 2 of 3 island boards). Nine of the 10 mainland boards’ OPAT services included both supported hospital discharge and admission avoidance pathways, with one admission avoidance only for SSTIs as part of an ambulatory care service. One island board operated some OPAT activity within a hospital-at-home service, and the remaining island board on the group reported ad hoc outpatient IV therapy without overarching governance processes. One mainland health board not included within SAPG OPAT reported ad hoc outpatient IV therapy without overarching governance processes.

### Development of the OPAT SSTI pathway

Using the established clinical pathway, SAPG OPAT developed a final OPAT SSTI pathway for adults (18 years or older). A National Early Warning Score (NEWS2) of 0 or 1 was agreed for inclusion, although higher NEWS scores could be included based on local experience and expertise. The pathway was agreed suitable for use within a patient group direction (PGD)^8^ by qualified specialist OPAT nurses and by independent prescribers. The pathway emphasized clinical assessment and consideration of alternative pathologies. Given favourable OPAT experience in established services, daily IV ceftriaxone 2 g was agreed as first-line treatment when an IV route was indicated, with IV daptomycin or IV teicoplanin as alternatives in case of a β-lactam allergy or concerns regarding risk of *Clostridioides difficile*. The pathway stipulated daily attendance to optimize opportunities for IVOS to the agreed oral agents flucloxacillin or doxycycline (well established in both primary and secondary care guidance). Single-dose IV dalbavancin with review 1 week later was included as an alternative if the patient was unable to attend daily for review due to logistical or health or behavioural barriers (Figure 1a and b).

**Figure 1.**
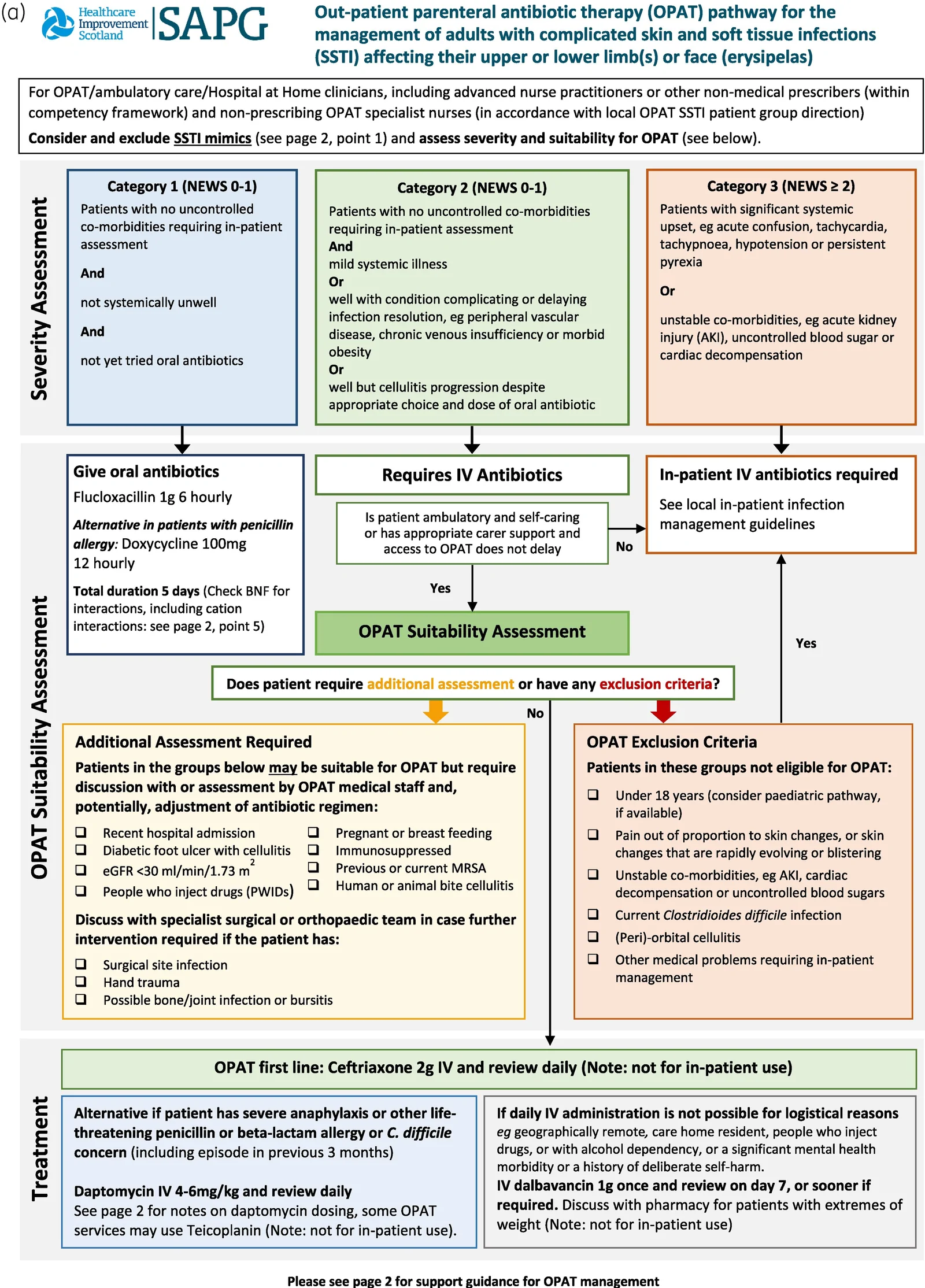

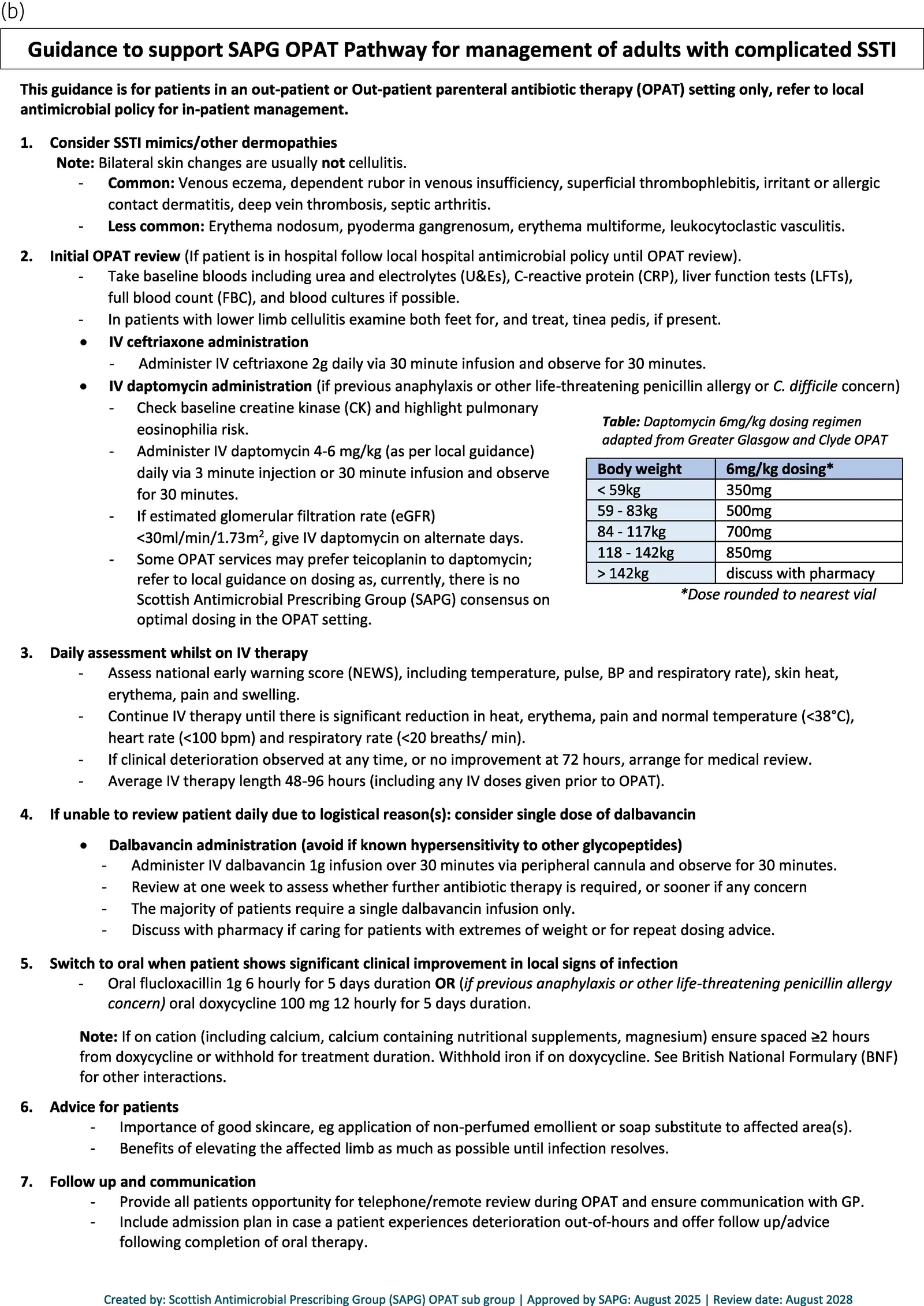
(a) The SAPG OPAT SSTI pathway. (b) Guidance to support the SAPG OPAT SSTI pathway.

SAPG OPAT agreed the pathway, which was reviewed and ratified by an SAPG committee. It was then shared with the Scottish Area Drugs and Therapeutics Committee Consortium, to support formulary processes, and with board Antimicrobial Management Teams (AMTs) and medical and pharmacy directors. The final pathway was published on the SAPG website in June 2022. Health board teams were provided with editable versions of the SAPG OPAT SSTI pathway. Review of the pathway in 2025 by SAPG OPAT and the SAPG committee resulted in only minor word changes for clarification.

### Survey of OPAT services

In February 2025, nine boards reported fully adopting or adapting the pathway: seven were within mainland OPAT services, one was in an ambulatory care service integrated into a limited OPAT service in a mainland board, and in one island board it was within hospital at home. Logistical barriers and lack of staff were cited as reasons for not implementing the SSTI pathway in the two remaining mainland boards’ OPAT services. The remaining three health boards only had ad hoc OPAT provision. Seven of the nine full OPAT services stated they managed infections in PWIDs, two services reported implementing PGD to support the pathway, six of nine services had nurse independent prescribers and six had clinical pharmacy independent prescribers. Five of the nine OPAT services were at least partially supported by hospital-at-home services.

In the April 2026 survey, one further mainland board had adopted the pathway as part of a comprehensive OPAT service, and one additional island board had adopted it as part of a hospital-at-home service.

By April 2026, 11 territorial boards had adopted the pathway across approximately 28 separate acute services; 8 in NHS Greater Glasgow and Clyde (three OPAT clinics and two ambulatory care units for full supervision of care and availability across three emergency departments); 5 in NHS Lothian (two OPAT clinics, two emergency departments and one medical admissions unit); 6 in NHS Highland (four acute hospital sites, one rural general hospital and one hospital-at-home service) and 2 in NHS Grampian (one OPAT clinic and one ambulatory care unit). Remaining boards reported the pathway active via a single OPAT or hospital-at-home service. The pathway was available across a population of approximately 5 million, or 91% of the Scottish adult population. Three boards (one island and two small mainland boards) reported ongoing OPAT activity without formal pathway adoption (Figure 2).

**Figure 2.**
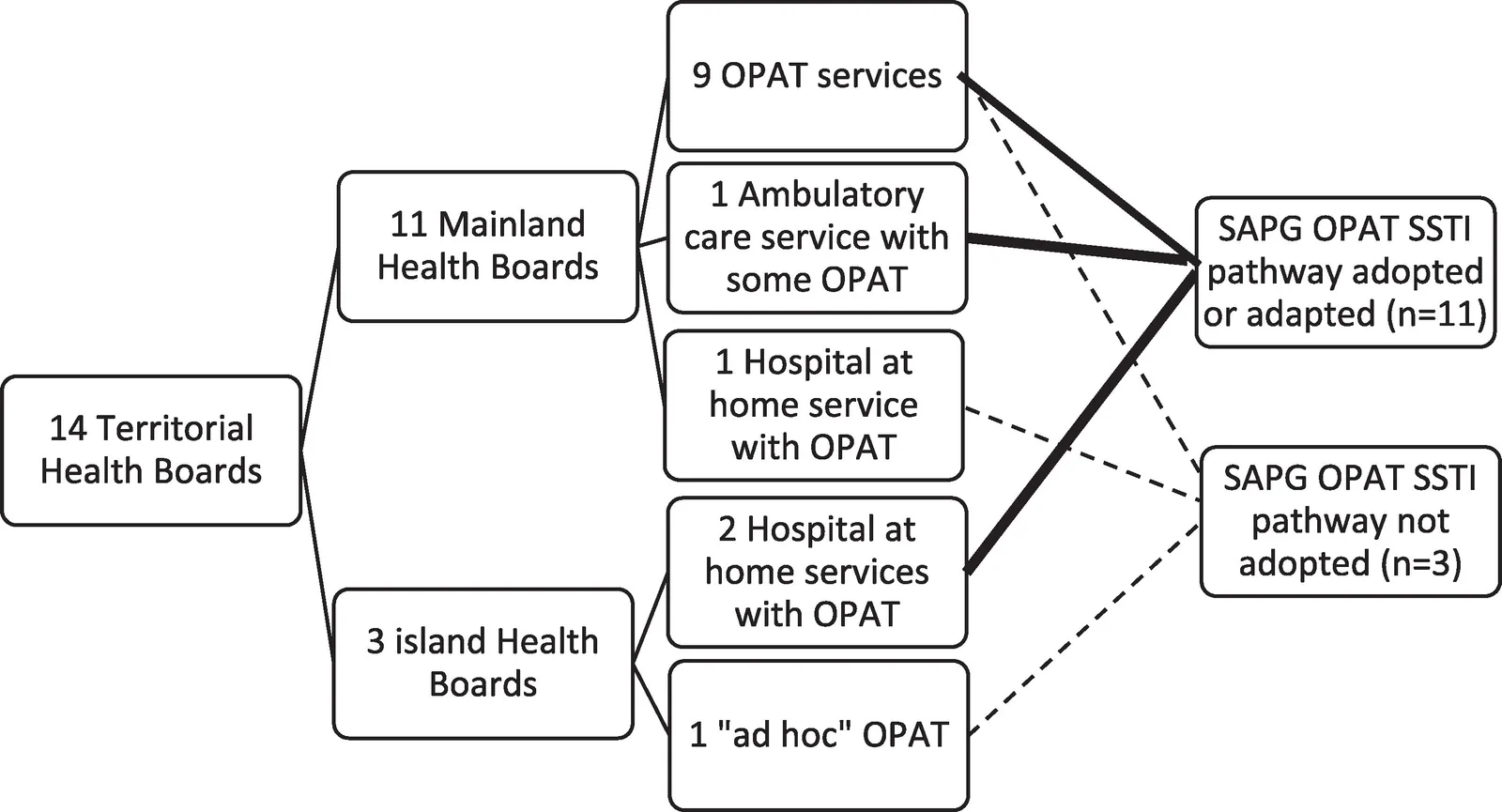
Adoption of the SAPG OPAT SSTI pathway in NHS Scotland (April 2026).

## Discussion

The NHS is under pressure with increasing admissions, longer emergency department waiting times,^11^ impaired patient flow and increased bed occupancy.^12^ Pathways to reduce attendance and support admission avoidance are emphasized in both the NHS England 10-year plan^13^ and the NHS Scotland operational improvement plan.^14^ While efficiency is important it is also essential that care is delivered equitably and AMS principles are maintained across care with particular attention to minimizing use of unnecessary IV antimicrobial therapy.

Cellulitis is a common reason for acute presentation and attendance at adult emergency departments, with an estimated >100 000 admissions per year in England^15^ and an accumulated length of stay of 73 470 days or approximately 6 days per episode in Scotland.^12^ There is variation in length of stay and implementation of timely IVOS in hospitals, and specialist nurse-led cellulitis pathways in Scotland have demonstrated that the duration of IV therapy is significantly shorter and less variable in the OPAT setting.^8,9^

The motivation to develop a national OPAT pathway for SSTIs was to address variation in service provision across a diverse and geographically dispersed population and to embed key AMS principles into care delivery while not compromising access to care for more vulnerable groups.^3^ A well-established pathway was adapted and consensus reached across a multidisciplinary group representing the majority of health boards in Scotland. *C. difficile* risk in association with ceftriaxone is low in the OPAT setting.^16^ However, alternative treatments were made available for those patients assessed locally as at higher risk. This local to national engagement and flexibility to allow for local adaptation was key to successful uptake and implementation across Scotland. The needs of those working across large, remote and rural populations where daily review would not be feasible was accommodated with the inclusion of long-acting dalbavancin. Dalbavancin allowed for the pathway to provide more equitable care, including improved access to other more vulnerable patient groups unable to be seen daily, including PWIDs.

The pathway is now active in 11 of Scotland’s 14 territorial health boards, and within those boards the pathway is deployed across multiple acute services. Discussions are ongoing to establish the pathway in the remaining health boards where OPAT practice is less developed or resource limited. Further work is planned to evaluate and compare antibiotic use between services, to more fully evaluate the impact of the pathway on admission avoidance and to develop other clinical pathways.^17^
